# Non invasive palliation for advanced pancreatic cancer using high intensity focused ultrasound

**DOI:** 10.1186/2050-5736-2-S1-A2

**Published:** 2014-12-10

**Authors:** Michele Rossi, Riccardo Naspetti, Ernesto Mazza, Paolo Boninsegni

**Affiliations:** 1 Azienda Ospedaliero universitaria Careggi – Firenze, Italy

## Background

A majority of patients (85-90%) diagnosed with pancreatic cancer has advanced disease at the time of diagnosis with a dismal prognosis. Pain is a common symptom in patients (60-90%) with advanced disease. Pain is neuropathic and inflammatory, resulting from both tumour expansion and tumour invasion of the celiac and mesenteric plexus. Opioids have side effects and may not be sufficient while celiac plexus neurolysis is an invasive procedure, despite the questionable results reported. Currently, HIFU (high intensity focused ultrasound) an emerging procedure completely no invasive, has been gaining wide acceptance not only for treating neoplasm but for symptom’s relief also.

## Materials and methods

A 76 year old lady was referred to us with an advanced head-body adenocarcinoma of the pancreas with vascular encasement of the superior mesenteric artery and vein. She was jaundiced (bilirubin of 16 mg/dL) and complained of back pain consistent with tumor-related pain with a visual analogue scale (VAS) of 9/10 and daily opioids assumption. Endoscopic placement of a metal stent followed by HIFU treatment was considered the best suitable option.

## Results

A single step HIFU procedure was performed after an endoscopic retrograde cholangiopancreatography (ERCP) and biliary metallic stenting. Total treatment time was 1 h and 50 min, with a sonication time of 60 min. Treatment power used ranged between 400 and 150 W, although mainly higher intensity energies were used. Total ablated volume was 80%. No complications occurred and total hospitalization lasted one day. Amylase and bilirubin level showed no significant elevation in the 7 days post procedure. Pain was observed at discharge with a VAS of 4/10, while no further referred after 7 days. Patient was pain free at first and third month with no need of oral analgesic drugs as CT scan confirmed adequate response with absence of perfusion on imaging. Unfortunately, due to tumor progression patient died 4 months later.

## Conclusion

Palliation has to be achieved while causing as little harm as possible. We proved HIFU to be successful in symptoms palliation in a patient with advanced pancreatic neoplasm. We recommend HIFU treatment introduction and development in several oncologic diseases, not only for therapeutic purposes , but also to improve patient quality of life.

